# Gut commensal bacteria-derived polysaccharide sub-micron particles induce antigen-specific, tolerogenic responses

**DOI:** 10.3389/fimmu.2025.1599480

**Published:** 2025-09-25

**Authors:** Rian Harriman, Allen B. Tu, Zhenyu Wang, Nicolas Radoc, Hamilton Kakwere, Jamal S. Lewis

**Affiliations:** ^1^ Department of Biomedical Engineering, University of California, Davis, Davis, CA, United States; ^2^ J. Crayton Pruitt Family Department of Biomedical Engineering, University of Florida, Gainesville, FL, United States

**Keywords:** polysaccharide a, sub-micron particles, immunomodulatory, dendritic cells, T cells, regulatory

## Abstract

Roughly 10.8% (>26 million) Americans suffer from food allergies (FA) which, in severe cases, can be life threatening. Oral immunotherapy (OIT) offers a promising allergen-specific approach in the management of FA. However, due to risk of anaphylactic shock, there are significant concerns regarding its safety and must be carried out in the hospital under careful supervision by clinicians. These concerns may be addressed through delivery of the allergen in engineered nanoparticulate packages which may additionally improve therapeutic efficacy. Polysaccharide A (PSA), a commensal-derived molecule produced by the gut-symbiont *Bacteroides fragilis*, has shown tremendous potency in ameliorating inflammatory diseases in various mouse models by eliciting tolerogenic T cell activity. The tolerogenic capacity in combination with its polymeric structure makes PSA an intriguing biomaterial for the formulation of tolerogenic sub-micron particles. We hypothesized that encapsulation of protein antigen within PSA sub-micron particles (SMPs) would provide a particle platform capable of inducing robust specific tolerogenic responses for safer treatment of FA. In this body of work, we demonstrate the successful fabrication of tolerance-inducing sub-micron particles using the commensal-derived molecule, PSA. We reveal that PSA sub-micron particles can be easily loaded with ovalbumin (OVA), a surrogate for protein allergens, resist degradation in gastric fluid, and induce OVA-specific tolerogenic responses. Taken altogether, our findings give credence that PSA SMPs are ideally suited for OIT applications. Moreover, this study demonstrates that PSA SMPs have the potential to serve as a “plug and play” system capable of inducing specific tolerance to any encapsulated antigen.

## 1 Introduction

About 10.8% (>26 million) Americans suffer from potentially life-threatening food allergies (1). Allergic individuals typically manage their conditions through strict food avoidance and/or the administration of antihistamine upon accidental exposure (2). Furthermore, the constant risk of accidental consumption can place a tremendous psychological toll on patients. Summarily, there is a significant unmet need for a more proactive approach for combating food allergies. Oral immunotherapy (OIT) offers a promising allergen-specific approach in the management of food allergies (FA) but still has significant shortcomings. Each dosage event carries the risk of accidentally triggering anaphylaxis and requires intensive medical supervision, especially at the start of OIT regimens (3). Moreover, long-term tolerance, or sustained unresponsiveness, after OIT is only established in 13-36% of patients (4–8). Thus, most patients must undergo maintenance dosages for several months, years, and possibly indefinitely to retain their desensitized immunological state (9). This degree of compliance to maintain desensitization may be unsurmountable for many patients and negate the benefits of current OIT strategies. Although tremendous strides have been made in the implementation and characterization of OIT, next-generation OIT strategies must address the substantial shortcomings in safety, efficacy, and compliance.

**Figure 1:**
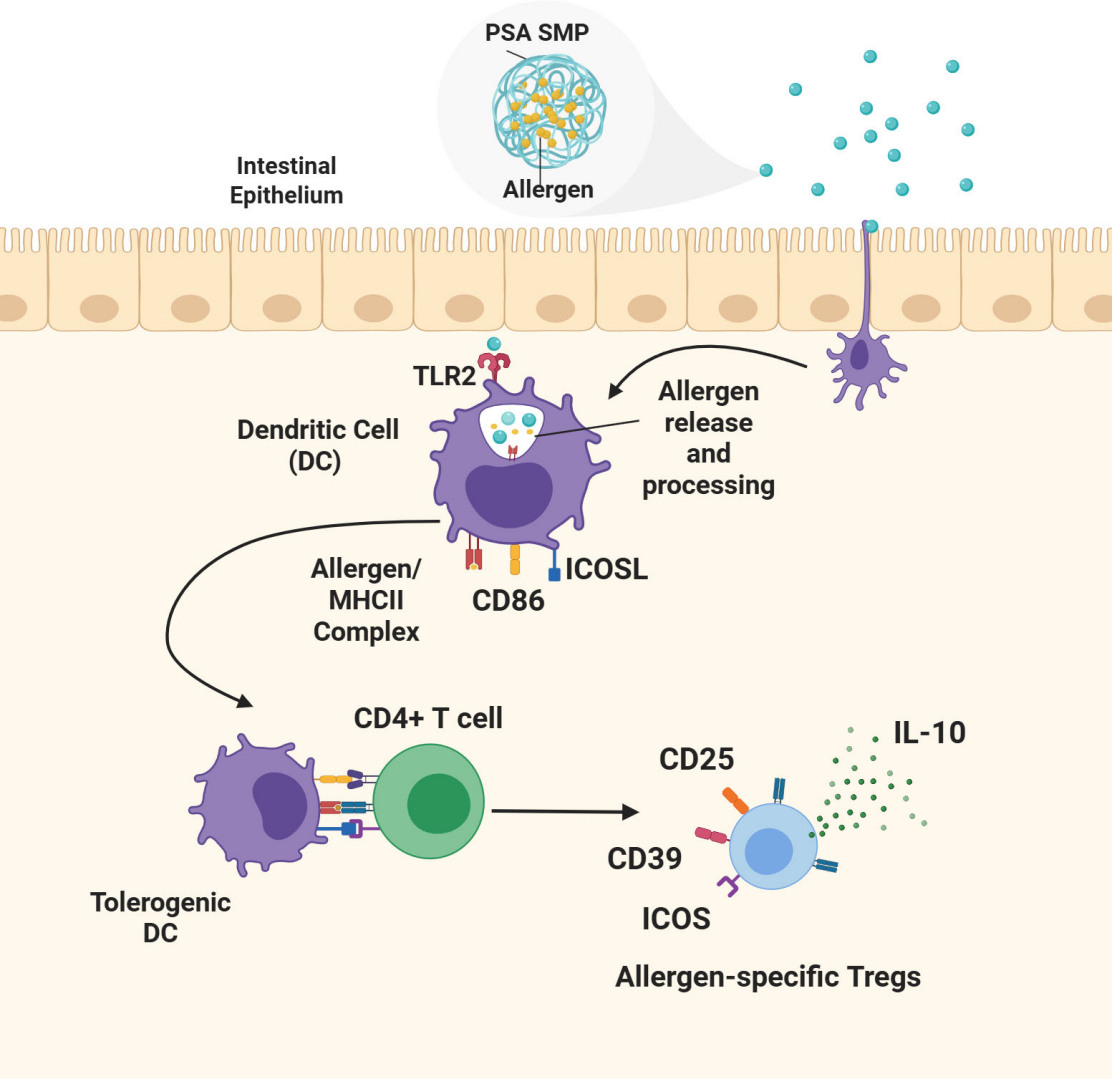
Proposed mechanism of oral tolerance induction by allergen-encapsulated PSA SMPs. Allergen-encapsulated PSA SMPs are taken up by dendritic cells (DCs) inducing their differentiation to a tolerogenic phenotype characterized by the upregulation of ICOSL and CD86. The PSA SMPs are degraded within the dendritic cell, releasing the allergen for processing and loading onto the MHCII complex and presentation to CD4 T cells. PSA-trained DCs mediate the differentiation and expansion of allergen-specific T regulatory cells that promote immune protection against the encapsulated allergen through the upregulation of CD25, CD39, ICOS, and IL-10 secretion.

PSA, a tolerogenic polysaccharide found in the capsule of the gut commensal *Bacteroides fragilis*, has been denoted as the “archetypical” microbial symbiotic factor for its role in gut homeostasis and protective properties against inflammatory diseases both inside and outside of the gut (10). Its protective properties have been tied to its ability to induce IL-10-producing T regulatory (Treg) cells (10). Interestingly, several clinical studies indicate that sustained unresponsiveness after OIT is correlated with increased Treg cell populations, suggesting that combination of PSA with OIT may enhance efficacy (5–7). Moreover, physically coupling PSA’s immunomodulatory effects with allergen delivery may further augment OIT by directly mediating the induction of allergen-specific Treg cells. Thus, we hypothesize that the shortcomings of OIT can be addressed through the encapsulation of allergen within PSA SMPs due to their ability to deliver allergen and induce tolerogenic dendritic cell differentiation simultaneously. PSA-trained DCs process and present the encapsulated allergen to T cells which ultimately leads to the expansion of allergen-specific T regulatory cells and immune tolerance towards the delivered allergen (**Figure 1**). Here, we demonstrate that gastric-stable PSA-SMPs loaded with ovalbumin (OVA), a surrogate for protein allergen, can be processed and presented by dendritic cells to CD4+ T cells and induce their expansion and differentiation to a tolerogenic phenotype characterized by CD25, CD39, ICOS, and IL-10 upregulation. The unique tolerogenic and physiochemical properties of PSA SMPs make them ideally suited for OIT applications.

## 2 Materials & methods

### 2.1 Culture and growth of bacteroides fragilis (ΔmpiM44)

Brain heart infusion (BHI) broth (Research Products International) was dissolved in de-ionized water and autoclaved. Hemin and gentamycin were added aseptically into a working concentration of 5 µg/ml and 50 µg/ml, respectively, to make complete BHI media. Individual colonies of *Bacteroides fragilis* strain ΔmpiM44 grown on sheep blood agar plates were picked and transferred to 3ml of complete BHI starter cultures and incubated overnight at 37°C in an anaerobic chamber (Shel Lab, Bactron II). The starter cultures were used to inoculate glass bottles containing 500ml of BHI media and subsequently incubated for 5 days. The cultures were then combined and pelleted for further processing (11).

### 2.2 PSA isolation and chemical characterization

Bacterial pellets were suspended in water and sonicated vigorously to ensure a homogenous suspension. The suspension (200ml) was then heated to 65°C and added to an equal volume of preheated phenol in a beaker. The resulting mixture was transferred to a hot plate and stirred vigorously. Once the temperature reached 65°C, the temperature and stirring was maintained for an additional 35 minutes. The mixture was transferred to conical tubes and allowed to cool overnight at 4°C. The following day, the conical tubes were centrifuged at 10,000 x g and the water phase extracted. The water phase was then washed with 1:1 diethyl ether and concentrated using a rotary evaporator. The concentrate was transferred to a 10 kDa MWCO dialysis bag (Fisher Scientific) and placed in bulk water overnight and then lyophilized. The lyophilized material was then resuspended in PBS and treated with RNase (Millipore Sigma) and DNase (Millipore Sigma) at 0.33 mg/ml and 0.07 mg/ml, respectively, overnight at 37°C. The following day, proteinase K (Millipore Sigma) was added at 0.33 mg/ml and the mixture incubated for an additional day. The mixture was then centrifuged and the supernatant dialyzed. After 2 days of dialysis, the mixture was lyophilized. The resulting lyophilized material was resuspended in 1% sodium deoxycholate, 50 mM Glycine, 10 mM EDTA buffer and introduced to an SEC column containing Sephacryl S-300 HR Resin (Millipore Sigma). Fractions of 10ml were collected and assessed for PSA content using emerald 300 staining kit (ThermoFisher Scientific) following manufacturer’s instructions. LPS-free fractions containing PSA were consolidated and transferred to 10 kDa dialysis bags for dialysis in 0.5x PBS. The PSA was dialyzed for four days in 0.5x PBS, followed by three days in purified water. After a total of 7 days of dialysis, the bag’s contents were transferred to conical tubes and lyophilized to obtain purified PSA. The purity and structure were assessed by nuclear magnetic resonance (NMR) (800MHz Bruker Avance III).

### 2.3 PSA sub-micron particle fabrication

Polysaccharide A SMPs were fabricated using water/oil emulsification with glutaraldehyde crosslinking. Briefly, 4 mg of PSA with or without OVA (Invivogen, EndoFit Ovalbumin) (10:1 PSA to OVA by mass) was resuspended in 1ml water and added dropwise to 40ml of light paraffin oil and span80 (100:1 oil to span80 by volume) at a rate of 400 µl/min using a syringe pump under constant homogenization. A volume of 200 µl of 85 mg/ml glutaraldehyde in water was added dropwise at a rate of 3.3 µl/min using a syringe pump while stirring. Stirring continued for 24h until the turbidity subsided. Once the solution became clear, 140 µl propylamine was added and stirred overnight to quench residual glutaraldehyde. The oil phase was removed with two hexane washes followed by two ether washes and two water washes. The particles were sonicated briefly between each wash to ensure suspension. The particles were placed in 10 kDa MWCO dialysis bags for 4 days prior to use. To generate PSA to generate OVA-only sub-micron particles (OVA-SMPs), 4mg of OVA was processed using the same methods but in the absence of PSA.

### 2.4 Phenol sulfuric assay for polysaccharide quantification

A 1 mg/ml solution of PSA in water was serially diluted 2-fold dilution in a 96-well plate to make a standard curve. Polysaccharide sub-micron particles and controls (50 µl) were added to the plate in triplicate. 150 µl sulfuric acid was added to each sample, followed by 30 µl of 5% phenol immediately after. The plate was floated on near boiling water and incubated for approximately 10min until brownish-yellow coloration occurred. The plate was carefully removed, cooled and the absorbance of wells was measured at a wavelength of 490 nm, with 590 nm used as the ‘reference’ wavelength.

### 2.5 PSA sub-micron particle degradation

Simulated gastric fluid (SGF; Ricca chemical) was supplemented with pepsin (MP Biomedicals) according to manufacturer’s recommendation. PSA SMPs (100 µg) were resuspended in 1ml of SGF and incubated at 37°C for 1–3 h. To make nitric oxide, 50 mM diethylamine NONOate (Thomas Scientific) in PBS was sealed in a tube and incubated at 37°C overnight. NONOate spontaneously dissociates to nitric oxide at neutral pH (12). PSA SMPs (100 µg) were resuspended in 1ml of nitric oxide and incubated at 37°C for 1–3 h.

### 2.6 BCA assay for protein quantification

A MicroBCA Protein Assay kit (ThermoFisher Scientific) was used according to the manufacturer’s protocol for quantification of SMP-loaded protein. OVA at 1 mg/ml followed by a 2-fold serial dilution was used to make a standard curve. Unloaded (blank) SMPs were measured simultaneously and used for background correction. To calculate protein loaded into SMPs, absorbance from the unloaded (blank) SMPs was subtracted from OVA-loaded particles to obtain the “corrected absorbance”. This corrected absorbance is then applied to the standard curve to calculate the protein content in the particle.

### 2.7 Purpald assay for glutaraldehyde quantification

Purpald (4-Amino-3-hydrazino-5-mercapto-1,2,4-triazole; Sigma-Aldrich) was resuspended to 32 mM in 1M NaOH and mixed at 1:1 volumetric ratio with the samples. A 50% w/v glutaraldehyde in water solution followed by a 2-fold serial dilution was used as the standard. Absorbance at 320 nm was measured within 10 minutes after the addition of Purpald solution.

### 2.8 Physicochemical characterization of PSA sub-micron particles

SMPs were assessed for size and ζ-potential in diH_2_O using the Malvern ZetaSizer Nano ZS (Malvern Instruments, UK). Scanning electron microscopy images were captured using Philips/FEI XL30 SFEG Scanning Electron Microscope (SEM) for morphological and size assessment. Samples were prepared by sonicating PSA SMPs in water and drying on a silicon wafer overnight (16h). These samples were then sputtered with gold (5−10 nm-thick layer) and analyzed at 5.00 kV and 0.80 nA at magnifications ranging from 30x to 60,000x.

### 2.9 Experimental animals

Both male and female C57BL/6J and OTII (Strain #:004194, The Jackson Laboratory) mice were purchased from Jackson Laboratories. All animals were housed in specific pathogen-free environment conditions at the University of California, Davis TRACS facility and used according to the UC Davis Institutional Animal Care and Use Committee (IACUC).

### 2.10 Generation of bone marrow-derived DCs with FLT3-L

Dendritic cells were obtained from 8−12 weeks old male and female C57BL/6j or OTII mice in accordance with guidelines approved by the University of California, Davis, Animal Care and Use Committee (IACUC) using a 10-day protocol as described by Kakwere et al (13). Mice were euthanized by CO_2_ asphyxiation followed by cervical dislocation, and tibias and femurs were harvested for isolating bone marrow cells. The bone marrow cells were obtained by flushing the shaft of the long bones with a 25g needle using RPMI medium with L-glutamine and 25 mM HEPES (Mediatech, Manassas, VA) containing 1% fetal bovine serum (Mediatech) and 1% penicillin/streptomycin (Hyclone) (RPMI complete media) and mixed to make a homogeneous suspension. The suspension was then strained using 70 µm cell strainers (Becton Dickinson, NJ, United States), and cells were collected at 1800 rpm for 5min. The red blood cells (RBCs) were lysed with ACK lysis buffer (Lonza, Walkersville, MD) followed by centrifugation at 1800 rpm for 5min to recover leukocytes. Leukocytes were then resuspended in RPMI complete media supplemented with 100 ng/ml and 1X 2-Mercaptoethanol (Gibco™) and plated 3.5ml per well in a 6-well, tissue culture plate and incubated at 37°C, 5% CO_2_ incubator. On day 5, 1.5ml of medium from each well was replaced with 1.5ml fresh complete medium supplemented with 100 ng/ml FLT3-L and then incubated for 5 more days (10 days total). On day 10, these FLT3-L bone marrow-derived dendritic cells (fBMDCs) were used for *in vitro* experiments.

### 2.11 LDH cytotoxicity assay

On day 10, 1.25 x 10^4^ fBMDCs were seeded per well into a 96-well plate and treated with PSA SMPs at a dose of 5, 10, 25, 50, and 100 ug/mL for 48 hours. After 48 hours, a CyQUANT LDH Cytotoxicity Assay (Invitrogen) was performed per manufacturer’s protocol. Absorbance was determined at 490 nm and reference at 690 nm was subtracted. Percent cytotoxicity and percent viability were calculated as follows: %Cytotoxicity = (PSA SMP-treated DCs - media only)/(Lysed cells - media only) * 100. %Viability = 100 – %cytotoxicity.

### 2.12 TLR2 activation assay

HEK-Blue mTLR2 reporter cells (Invivogen) were treated with 25 µg/ml PSA, PSA SMPs, or OVA-loaded PSA-SMPs (PSA-OVA SMPs) for 16h in the absence or presence of blocking anti-mTLR2 antibody. Absorbance was measured at 625 nm. Specific mTLR2 activity was determined by subtracting absorbances of treatments without antibody by treatments with antibody and then normalized to the water control.

### 2.13 Dendritic cell culture and phenotypic analysis

fBMDCs from OT-II (C57BL/6j background) or C57BL/6j were plated in 24-well plates at 1.7 x 10^5^ cells/well in 500 µl complete media and incubated with 50 µg/ml PSA, PSA SMPS, or PSA-OVA SMPs for 2 days before flow cytometry analysis. Antibodies used for analysis include PE anti-mouse CD275 (ICOS Ligand) (clone HK5.3, Biolegend), APC anti-mouse CD86 Antibody (clone GL-1, BioLegend), Alexa Fluor^®^ 488 anti-mouse I-A/I-E Antibody (clone M5/114.15.2, BioLegend).

### 2.14 Antigen-specific co-culture assay and phenotypic analysis

CD4+ T cells were isolated from OT-II mice using STEMCELL CD4+ Isolation kit (STEMCELL Technologies, 19852) and incubated with fBMDCs in a 96-well round bottom plate in 200 µl (150,000 T cells and 25,000 fBMDCs per well). PSA, PSA SMPs, and OVA-loaded PSA SMPs were added to a working concentration of 50 µg/ml and incubated for 4 days before performing flow cytometry analysis and IL-10 ELISA (BD OptEIA™ Mouse IL-10 ELISA Set 555252). Cells were stained with Fc block (BD Bioscience, 553142) and LIVE/DEAD™ Fixable Near-IR Dead Cell Stain Kit (Thermo Fisher Scientific, L34976) prior to surface staining. Surface antibodies used include PE-Cy7 anti-mouse CD4 (clone GK1.5, BioLegend), PE/Dazzle™ 594 anti-mouse CD39 Antibody (clone Duha59, BioLegend), Alexa Fluor^®^ 700 anti-human/mouse/rat CD278 (ICOS) Antibody (clone C398.4A, BioLegend), and Brilliant Violet 421™ anti-mouse CD25 Antibody (clone PC61, BioLegend). The stained cells were analyzed using an Attune NxT Flow Cytometer (Thermo Fisher Scientific), collecting at least 10,000 events per sample. Data were processed using Attune software (Thermo Fisher Scientific). Singlets of live cell populations were gated based on LIVE/DEAD™ Fixable Near-IR Dead Cell Stain Kit (Thermo Fisher Scientific, L34976). Positive staining gates were established using fluorescence-minus-one (FMO) controls.

### 2.15 Statistical analysis

One-way ANOVAs were used for all experiments. Statistical significance was determined by *post-hoc* pairwise comparisons using Tukey tests. For pairwise comparisons, the means of each treatment group were compared. Differences were considered significant if p ≤0.05 using the Prism software (Version 9, GraphPad, La Jolla, CA).

## 3 Results

### 3.1 PSA isolation and structural analysis

**Figure 2:**
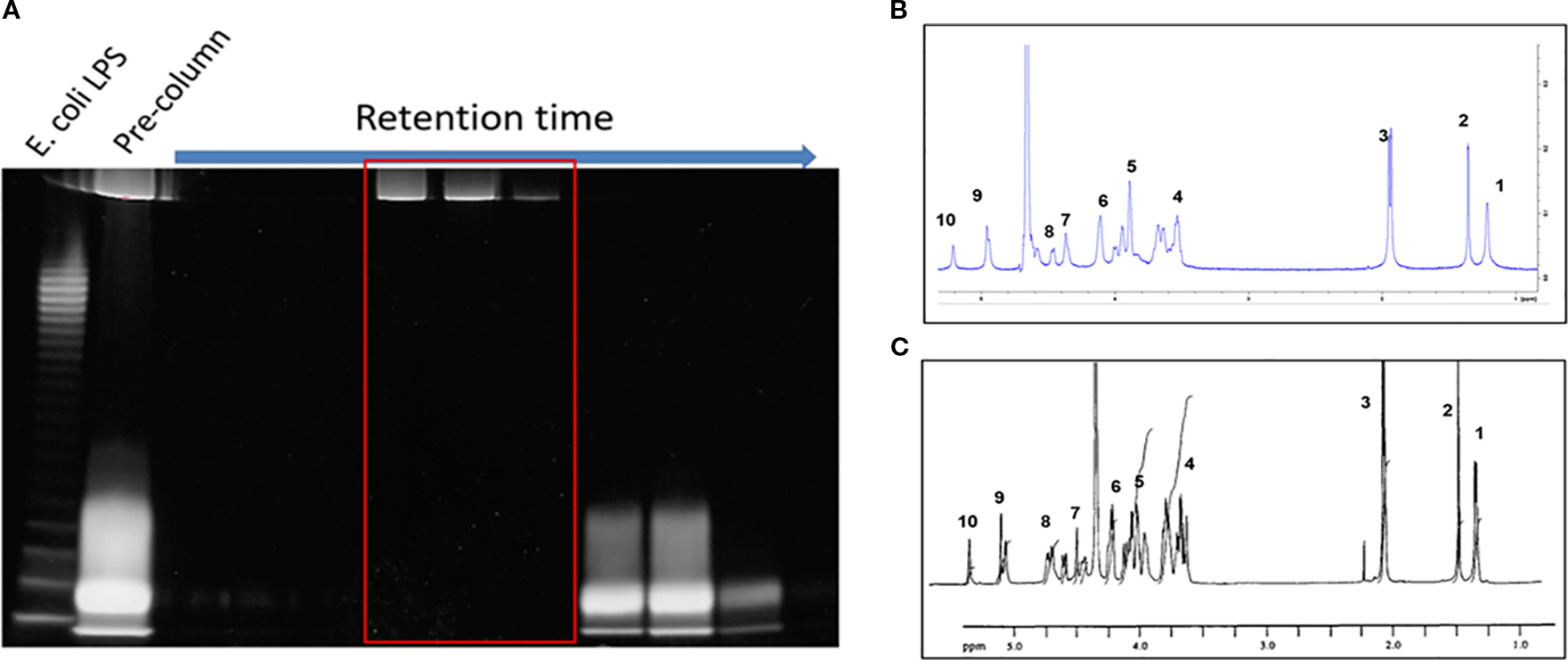
PSA isolation and purification. **(A)** 10ml fractions were collected sequentially from the SEC column and stained with Emerald 300. The larger PSA molecules (top band) are eluted earlier than the smaller LPS molecules (bottom band). The pre-column contains unseparated sample. LPS-free, PSA-only fractions (red box) are pooled, dialyzed, and quantified. **(B)** H^1^ NMR of isolated PSA vs NMR in literature (14). The number of peaks and peak shape of our PSA **(B)** matches previously published data **(C)**. The large peak between peaks 6 and 9 is likely hydrogenated solvent contamination.

To enable PSA’s tolerogenic potency, it is critical to separate this molecule from other capsular constituents including LPS, as well as proteins and nucleic acids found in the cell. PSA was purified from an engineered strain of *Bacteroides fragilis* (ΔmpiM44) that only expresses PSA, as well as LPS, in its capsule but is devoid of other capsular polysaccharides that do not have tolerogenic significance (11). Water-soluble components separated by hot phenol-water extraction were processed with DNAse, RNAse, Proteinase K and, contaminating nucleic and amino acids were subsequently removed using dialysis. LPS was then separated from PSA by size exclusion chromatography in sodium deoxycholate, which disaggregates LPS, so it runs as a relatively small molecule (~70kda) compared to the much larger PSA (~130kda) (**Figure 2A**). LPS-free fractions were then pooled and dialyzed in diluted PBS to remove sodium deoxycholate, and the purity and structure of isolated PSA was confirmed via nuclear magnetic resonance (NMR) (**Figures 2B, C**). Approximately 150 mg of polysaccharide was isolated from 20 L of liquid culture.

### 3.2 Fabrication and physicochemical characterization of PSA SMPs

**Figure 3:**
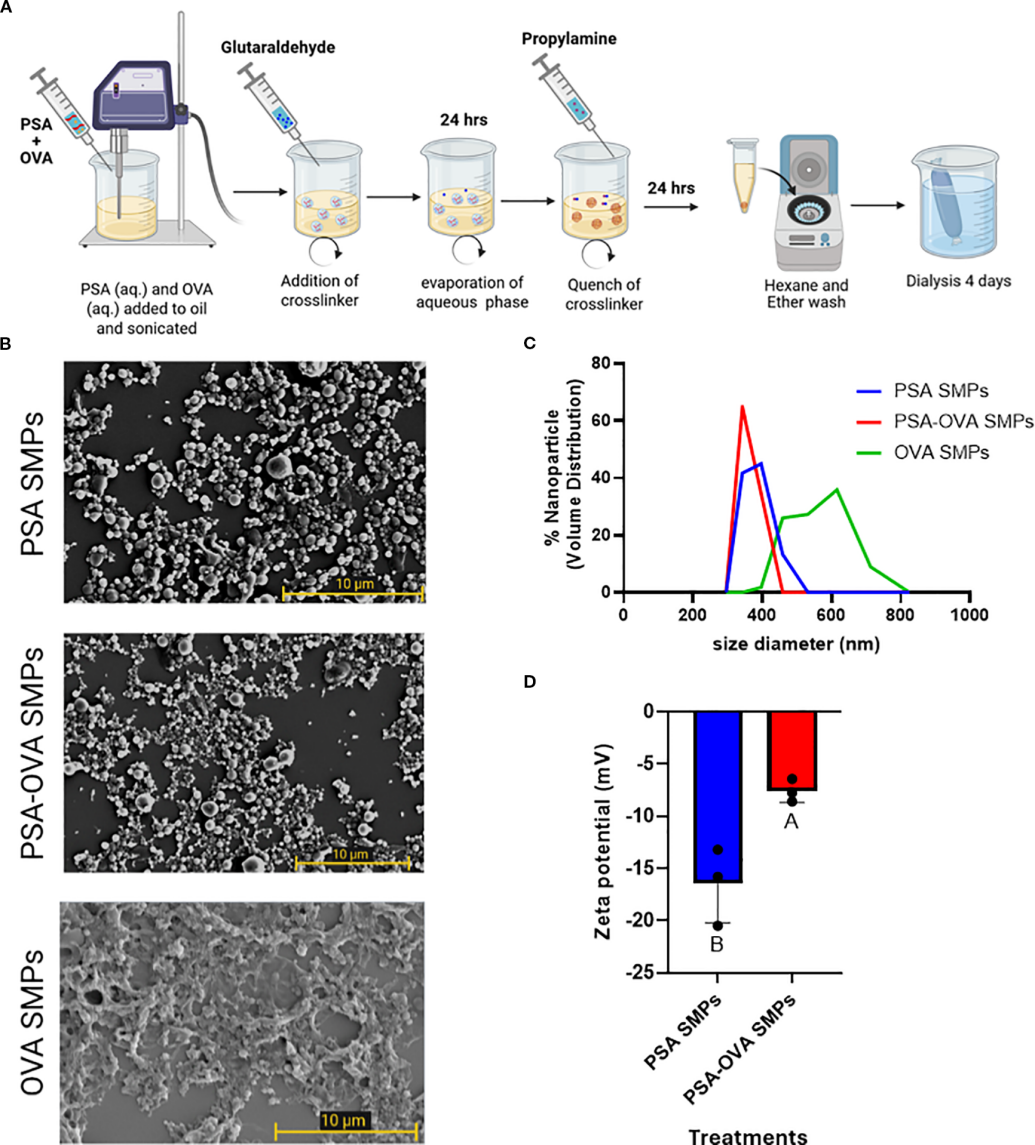
PSA sub-micron particle fabrication and characterization. **(A)** Schematic of the sub-micron particle fabrication process. **(B)** SEM imaging of PSA sub-micron particles and OVA-loaded PSA sub-micron particles revealed a spherical morphology, whereas OVA-only sub-micron particles are more filamentous and irregular. **(C)** DLS measurements showed that PSA SMPs and OVA-loaded SMPs have an average 350 nm in hydrodynamic diameter with a PDI of 0.818 and 0.672 (n=3), respectively, and **(D)** negative zeta potentials of -17.85mV and -7.1mV, respectively (p<0.05, n=3). Treatment groups sharing a letter are not significantly different, whereas groups with different letters are (p<0.05). An unpaired *t* test was used for statistical analysis via GraphPad Prism 9.

PSA SMPs were generated using a water-in-oil emulsification process with glutaraldehyde as a chemical crosslinker, which forms covalent bonds between amine groups found on each of PSA’s tetrasaccharide subunits (**Figure 3A**). OVA was used as a model allergen due to its routine usage in allergy mouse models, its availability, and for its applicability in antigen-specific *in vitro* assays (15–17). The size, morphology, and surface charge of the particles were characterized using dynamic light scattering, scanning electron microscopy, and zeta potential reading, respectively. PSA SMPs and OVA-loaded PSA SMPs (PSA-OVA SMPs) have a spherical morphology with an average hydrodynamic diameter of 350 nm. PSA SMPs and PSA-OVA SMPs particles had a negative zeta potential at -17.85mV and -7.1mV, respectively (**Figures 3B–D**). OVA-only particles were also fabricated as a control for downstream experiments. SEM imaging shows that OVA-only particles have a more filamentous and irregular structure.

### 3.3 PSA SMPs resist breakdown in simulated gastric fluid but are degraded in a nitric oxide solution

**Figure 4:**
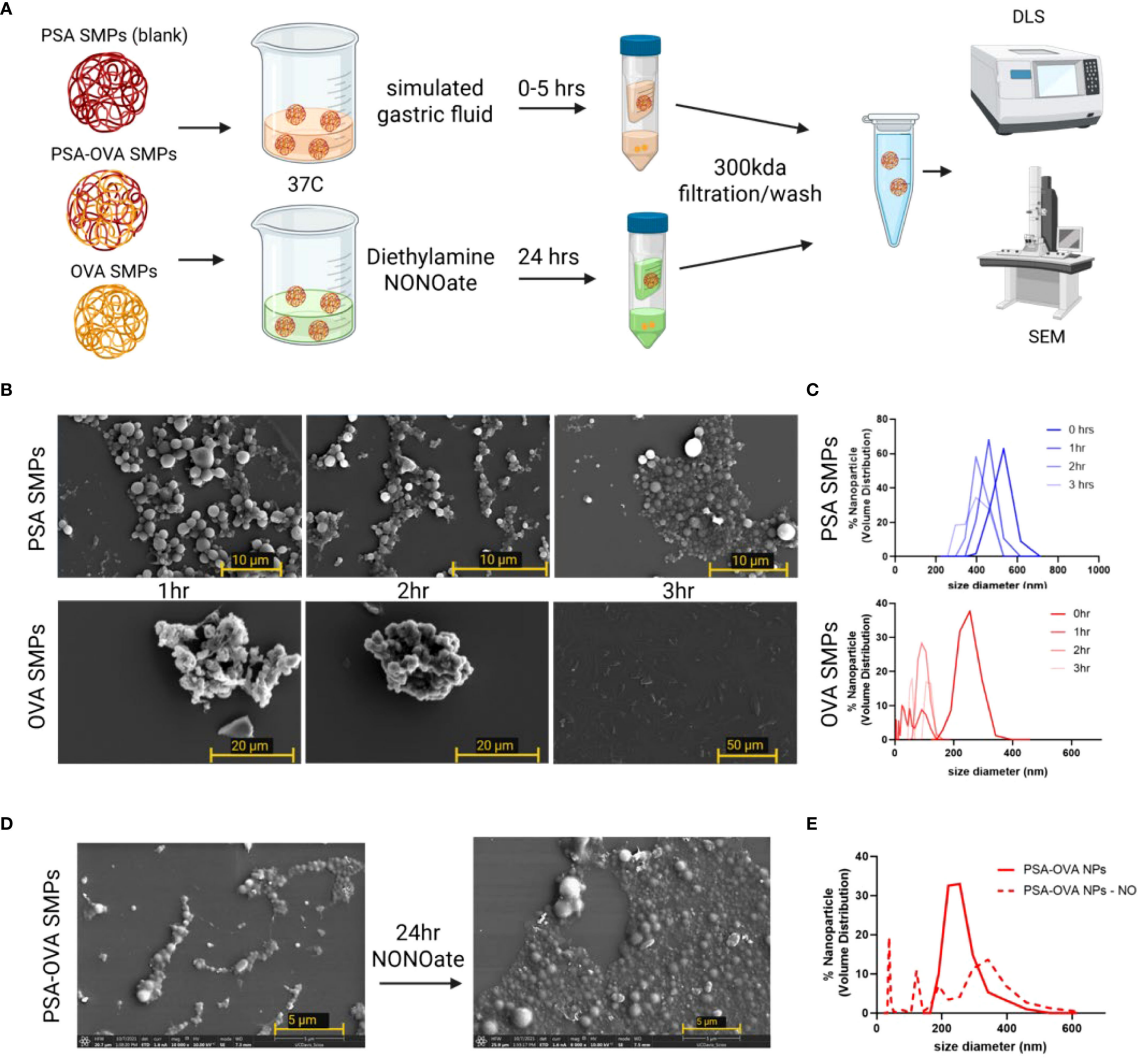
PSA sub-micron particle stability and degradation in simulated gastric fluid and nitric oxide. **(A)** Schematic for simulated gastric fluid and oxide treatment and analysis. **(B)** SEM and **(C)** DLS for PSA-OVA SMPs and OVA-only SMPs treated with simulated gastric fluid for various time periods. **(D)** SEM and **(E)** DLS of SMPs before and after 24hrs in a nitric oxide solution.

To test PSA SMPs suitability for oral administration, we first assessed whether the PSA SMPs could withstand harsh gastric conditions while maintaining their ability to be degraded in the phagosome. We used SGF with pepsin and dissolved nitric oxide (generated by NONOate) to simulate the degradative environments of the stomach and phagosome, respectively. Readouts for stability included changes in SMP morphology and size via SEM and DLS (**Figure 4A**). PSA-OVA SMPs were more resilient to SGF than OVA-only particles, as the latter was completely dissolved after the three-hour exposure period (**Figures 4B, C**). Investigators have found that depolymerization of PSA in the phagosome depends on the presence of nitric oxide (18). Therefore, we hypothesized that PSA-OVA SMPs will be degraded in the phagosome and release the packaged protein for antigen processing and presentation. Interestingly, there are clear morphological changes in PSA-OVA SMPs after exposure to nitric oxide, suggesting degradation of PSA sub-micron particles (**Figure 4D**). This is supported by the wider size distribution observed via DLS after nitric oxide treatment (**Figure 4E**).

### 3.4 PSA sub-micron particle quantification and loading efficiency

**Figure 5:**
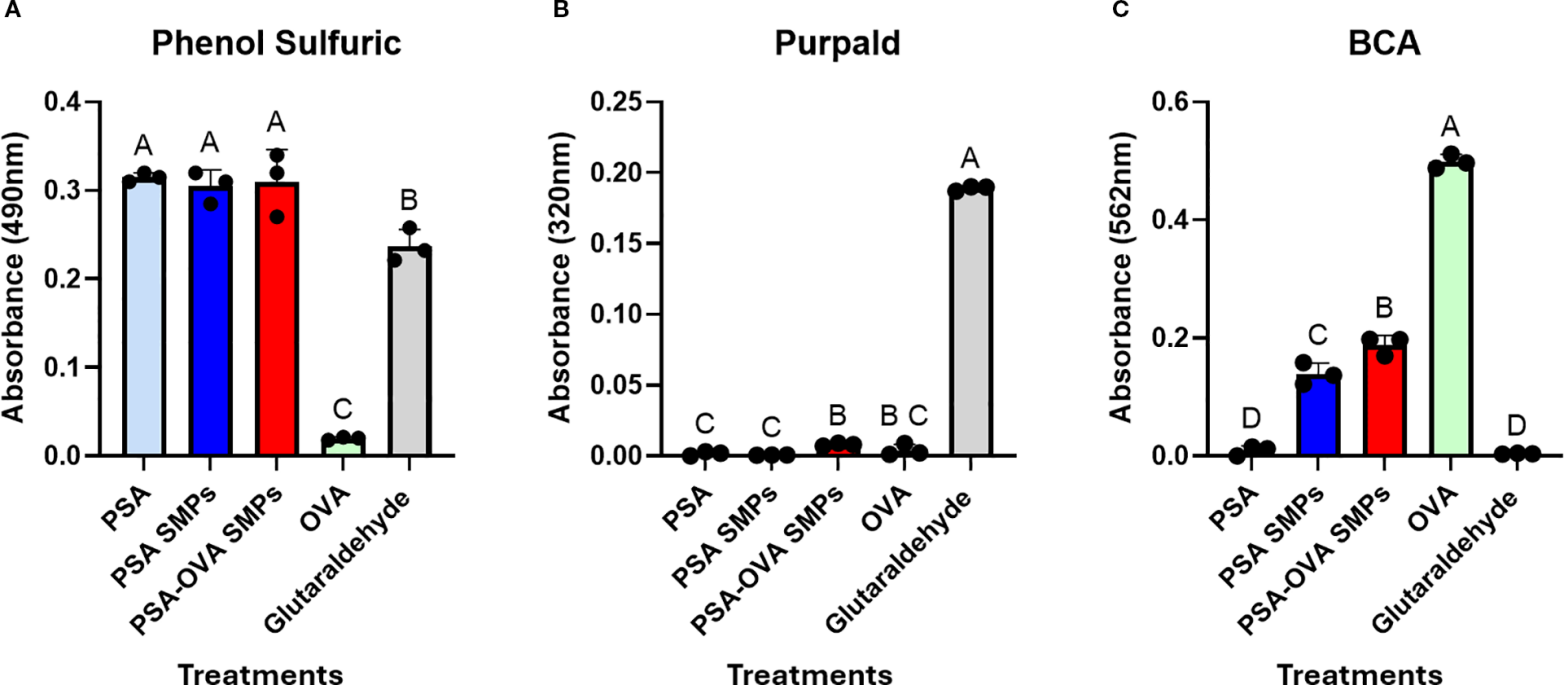
PSA-OVA SMPs composition. Soluble reagents, PSA SMPs, and OVA-loaded SMPs were normalized to 1 mg/ml and subjected to phenol sulfuric, BCA and Purpald assays to determine relative amounts of polysaccharide, protein, and glutaraldehyde respectively. **(A)** Phenol sulfuric assay detected polysaccharides and glutaraldehyde but not OVA (n=3, p<0.05). **(B)** Purpald assay revealed that active glutaraldehyde is undetectable in PSA sub-micron particle preparations (n=3, p<0.05). **(C)** BCA assay detected soluble and PSA-encapsulated OVA, but background absorbance is generated when PSA is formulated into particles (n=3, p<0.05). OVA within PSA-OVA SMPs can be quantified by subtracting background generated by blank PSA sub-micron particles prior to quantification using a standard curve. Treatment groups sharing a letter are not significantly different, whereas groups with different letters are (p<0.05). All statistical analyses were one-way ANOVAs corrected for multiple comparisons Tukey’s test via GraphPad Prism 9.

Normalized amounts (200 µg) of PSA, OVA, PSA SMPs, PSA-OVA SMPs were tested on the phenol sulfuric, BCA, and Purpald assays to evaluate the specificity of each assay and the relative polysaccharide, protein and glutaraldehyde, respectively, of each sample. The phenol sulfuric assay detects aldehydes, including glutaraldehyde (**Figure 5A**). However, due to propylamine quench and extensive dialysis during fabrication, residual glutaraldehyde is not present in particle preparations. This is also confirmed by the observation that free glutaraldehyde is not detected in PSA SMP preparations in the Purpald assay (**Figure 5B**). To quantify protein, we subjected the loaded and non-loaded particles to the BCA assay. We hypothesized that the small Cu^2+^ ions of the BCA assay would penetrate the PSA sub-micron particles and be reduced by the peptide bonds in the protein. The resulting Cu^+^ ions could then leach out of the particle and react with BCA molecules to induce color change. Interestingly, while soluble PSA doesn’t induce any color change in the BCA assay, unloaded PSA SMPs does show measurable background (**Figure 5C**). This could be due to nonspecific absorption of Cu2+ ions onto the zwitterionic PSA molecules which becomes measurable as PSA aggregates but remains undetectable when PSA is solubilized. To quantify protein in loaded particles, background absorbance from the blank particle was subtracted from the loaded particles prior to quantification using a standard curve. The encapsulation efficiency was measured to be 84% (n = 3), indicating that OVA makes up approximately 8% of the total mass of the PSA-OVA SMPs.

### 3.5 PSA and PSA SMPs bind TLR2 receptors

**Figure 6:**
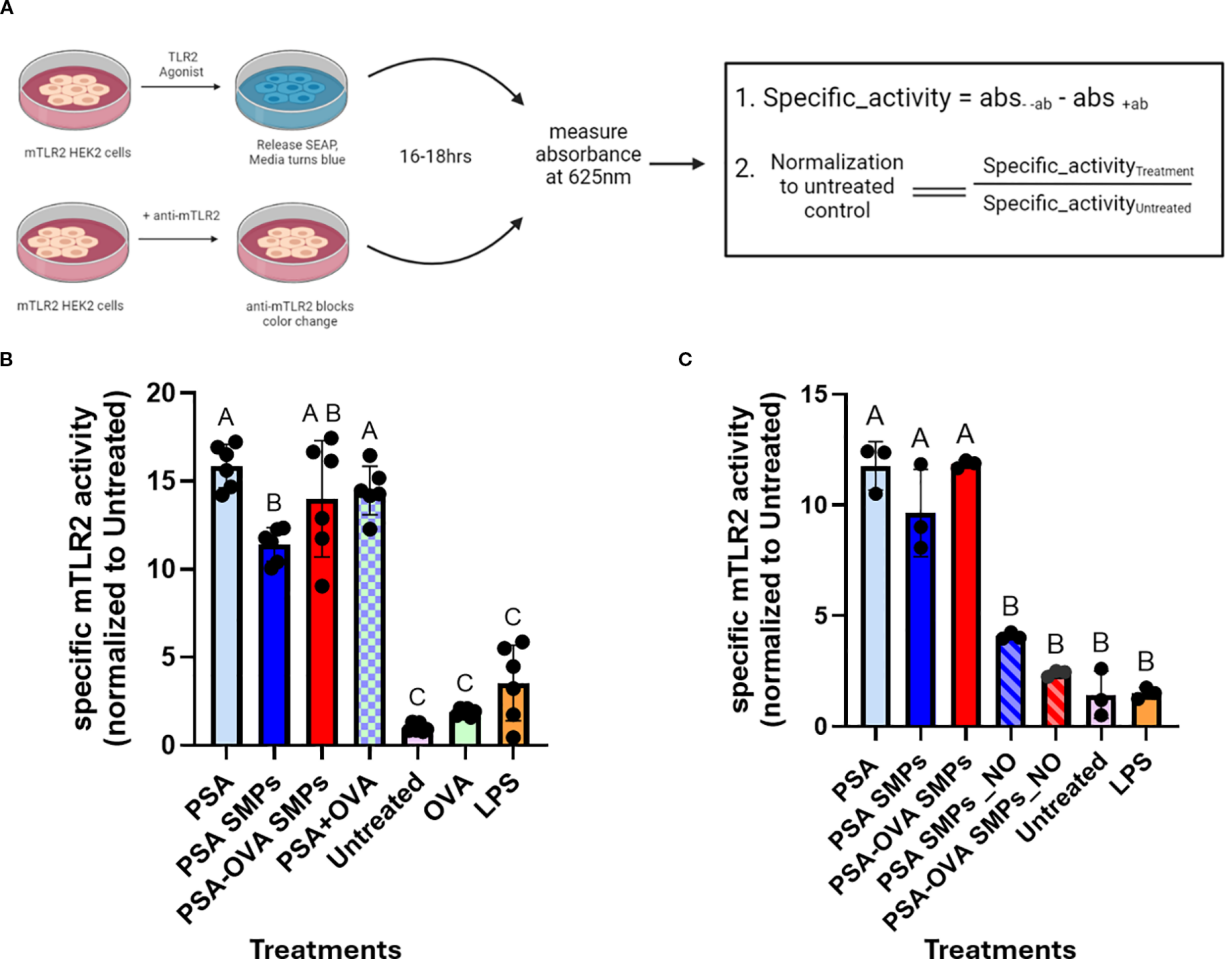
mTLR2 specific activity of PSA SMPs. **(A)** Schematic for specific mTLR2 assay. mTLR2 cells were treated in the presence/absence of blocking anti-mTLR2 antibody for 16hrs before measuring absorbance. The difference in absorbance between treatments without antibody and with antibody was calculated to find specific activity, which was then normalized to the untreated control. **(B)** Specific mTLR2 activity PSA SMPs or OVA-loaded PSA SMPs (PSA-OVA SMPs) were compared to soluble PSA and OVA either individually or in combination (n=6, p<0.05). **(C)** mTLR2 activity of the SMPs before and after nitric oxide treatment (n=3, p<0.05). Treatment groups sharing a letter are not significantly different, whereas groups with different letters are (p<0.05). Statistical analysis for experiments where n>3 were one-way ANOVAs corrected for multiple comparisons Tukey’s test, whereas experiments where n=3 utilized the Kruskal-Wallis test. All statistical analysis were performed using GraphPad Prism 9.

We investigated whether PSA SMPs maintained their ability to stimulate TLR2, as TLR2 activation by PSA is critical for initiating the transcription of genes involved in PSA processing and presentation, such as iNOS, MHCII, CD86 and ICOSL (10). To assess specific TLR2 activation by PSA SMPs, we utilized the HEK-Blue™ TLR Cells (InvivoGen) which are engineered to co-express murine TLR2 gene and an NF-κB-inducible secreted embryonic alkaline phosphatase (SEAP) reporter gene that can be quantified using the SEAP detection media. PSA, PSA SMPs, or PSA-OVA SMPs were incubated in the presence or absence of anti-mTLR2 blocking antibodies and the difference in absorbance was used to calculate the specific activity (**Figure 6A**). LPS produced an intensive color change with or without blocking antibodies, which indicates that LPS stimulation of the mTLR2 cells is not specific to TLR2. In contrast, mTLR2 cells incubated with OVA are not activated even in the absence of anti-mTLR2 blocking antibody, which indicates TLR2 is not activated by OVA. PSA, whether in particulate or soluble form, produced a >10-fold increase in specific activity compared to the untreated control, for mTLR2 stimulation was completely negated when anti-mTLR2 blocking antibody was included (**Figure 6B**). As expected, PSA sub-micron particles treated with nitric oxide had reduced capacity to activate the mTLR2 cells due to depolymerization of PSA and the breakdown of the particle (**Figure 6C**). These data suggest that sub-micron particle fabrication does not reduce PSA’s ability to stimulate TLR2 with high specificity.

### 3.6 PSA SMPs drive semi-mature, regulatory phenotype in fBMDCs

**Figure 7:**
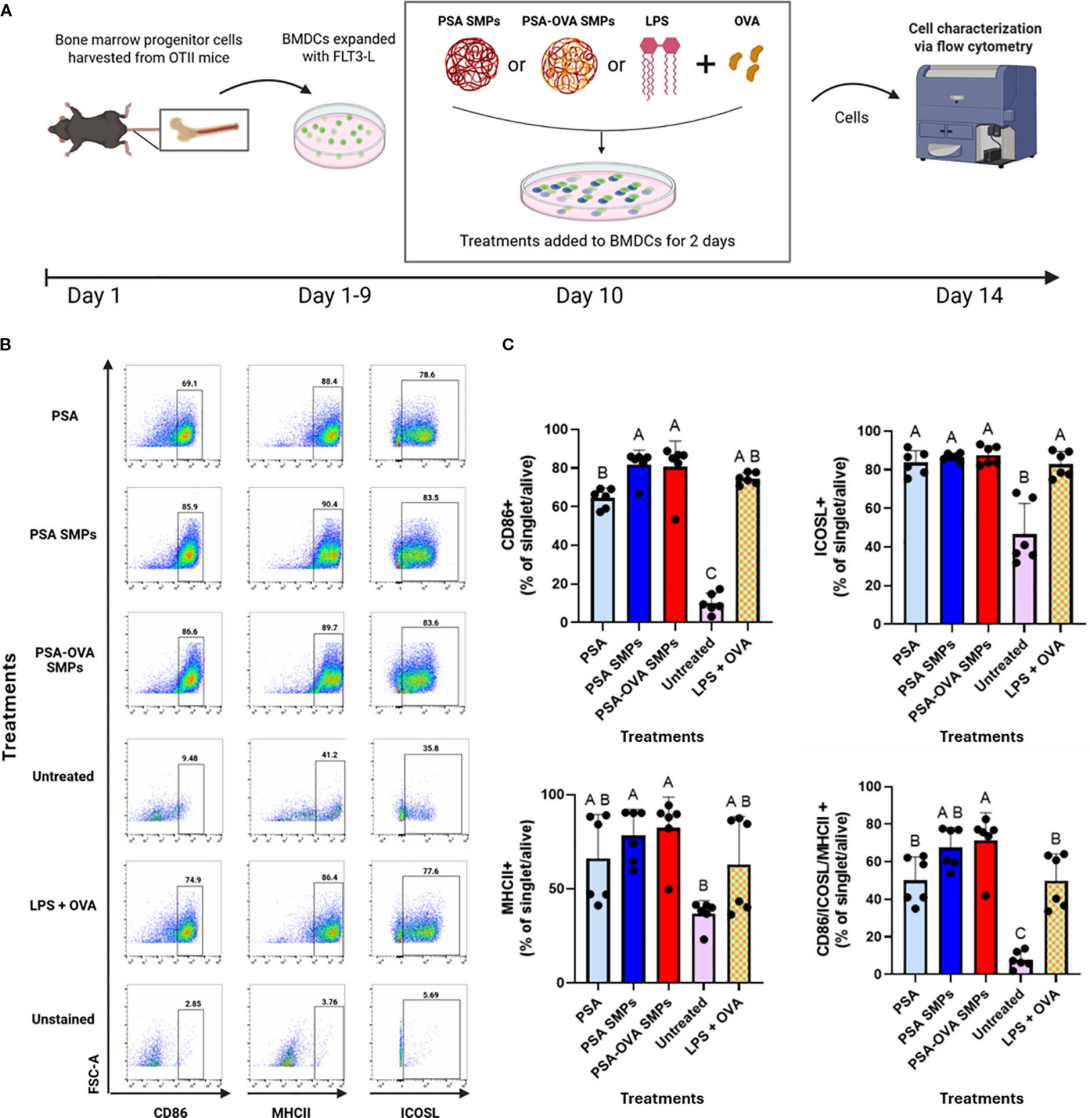
PSA SMPs drive a semi-mature and regulatory phenotype in fBMDCs. **(A)** Schematic of DC immunomodulatory analysis. BMDCs were treated with PSA, PSA SMPs, or OVA-loaded PSA SMPs (PSA-OVA SMPs) for 2 days and assessed by flow cytometry. **(B)** Representative dot plots of CD86, MHCII and ICOSL, of BMDCs after 2 days of their respective treatment. **(C)** Percent of BMDCs expressing CD86, MHCII, and ICOSL individually, and co-expressing all three markers (bottom right) after 2 days of their respective treatment (n=6, p<0.05). All statistical analyses were one-way ANOVAs corrected for multiple comparisons Tukey’s test via GraphPad Prism 9.

Current literature suggests that PSA interaction with surface receptors such as TLR2, trigger DCs to phagocytose and induce a tolerogenic phenotype that is characterized by an upregulation of MHCII, ICOSL, and CD86, in addition to antigen processing machinery such as iNOS (10). fBMDCs incubated with PSA SMPs (and relevant controls) for 2 days did not exhibit cytotoxicity (**Supplementary Figure 1**). Subsequently, PSA-OVA SMPs treated-fBMDCs were assessed by flow cytometry for phenotypical markers (**Figure 7A**). PSA, PSA SMPs, and PSA-OVA SMPs all induced high co-expression of CD86, ICOSL, MHCII. PSA SMPs and PSA-OVA SMPs have slight upregulation in co-expression over the LPS+OVA control (**Figures 7B, C**). PSA SMPs and PSA-OVA SMPs generate a similar phenotype as soluble PSA, suggesting that PSA immunomodulation of DCs is not altered when in particulate form.

### 3.7 PSA SMPs drive OVA-specific T cell modulation

**Figure 8:**
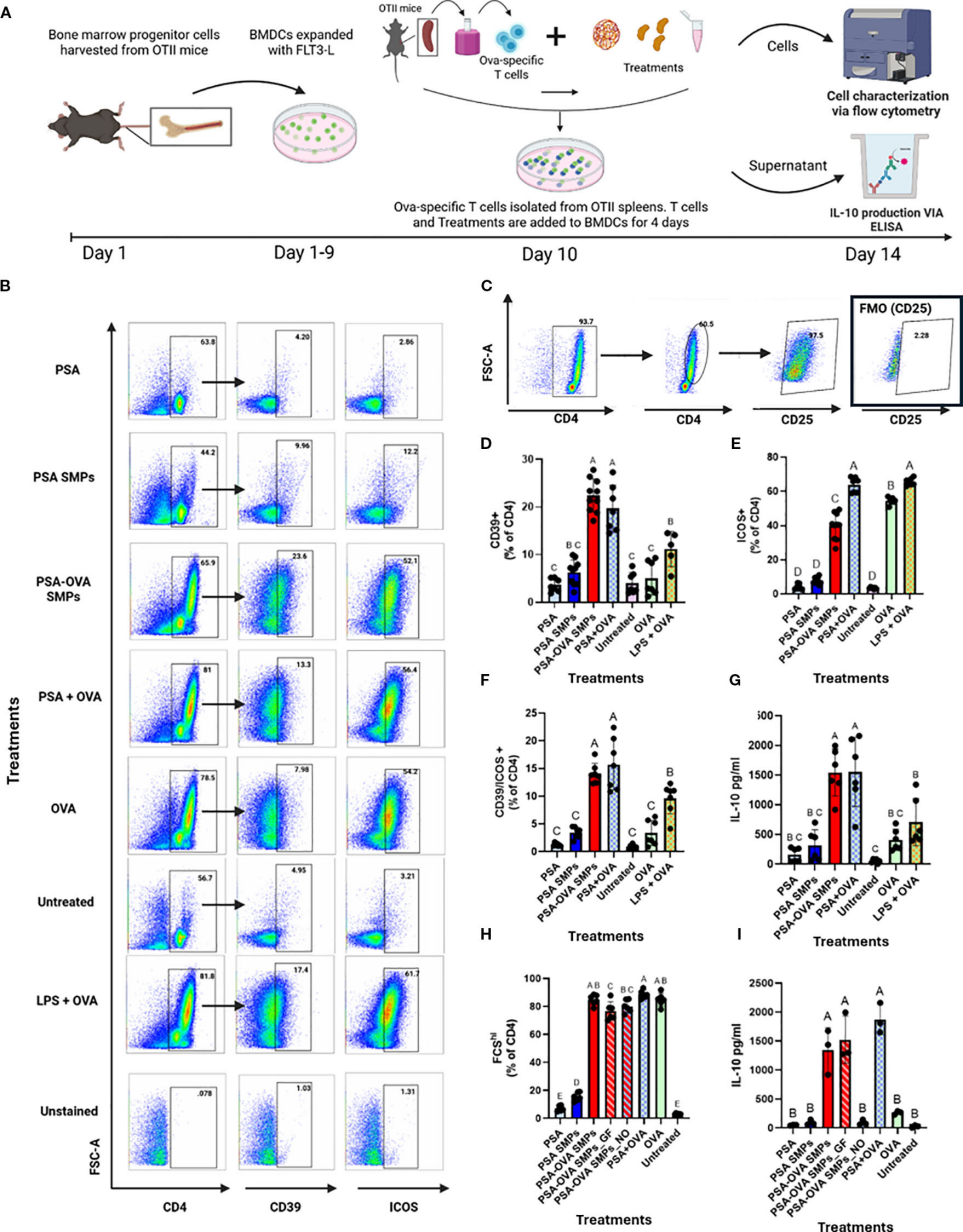
Antigen-specific T cell modulation. **(A)** Schematic for OVA-specific co-culture assay and downstream analyses. A co-culture experiment consisting of OT-II T cell and fBMDCs were co-incubated with PSA, PSA SMPs, or PSA-OVA SMPs for 4 days and assessed by flow cytometry and ELISA. **(B)** Representative dot plots of CD4, CD39 and ICOS on T cells after 4 days of their respective treatment. **(C)** Activated CD4+ T cells have increased size (FSC-A) and express CD25. **(D)** Percent of CD4+ T cells expressing CD39 and **(E)** ICOS individually, and **(F)** co-expression of CD39 and ICOS after 4 days of their respective treatment (n=6, p<0.05). **(G)** IL-10 in the supernatant of co-culture assay after 4 days of their respective treatment as determined by ELISA (n=6, p<0.05). **(H)** Percent of activated CD4+ cells in co-culture after 4-day exposure to OVA-loaded PSA sub-micron particles treated with gastric fluid (PSA-OVA SMPs_GF) or OVA-loaded PSA sub-micron particles treated with nitric oxide (PSA-OVA SMPs_NO) (n=6, p<0.05), and **(I)** IL-10 content in the supernatant as determined by ELISA(n=3, p<0.05). Statistical analysis for experiments where n>3 were one-way ANOVAs corrected for multiple comparisons Tukey’s test, whereas experiments where n=3 utilized the Kruskal-Wallis test. All statistical analysis were performed using Graphpad Prism 9.

To assess whether PSA SMPs could successfully deliver protein to DCs that is conducive to tolerogenic presentation, a co-culture assay consisting of fBMDCs, CD4 T cells derived from OT-II mice, and OVA-loaded PSA SMPs was performed and assessed by flow cytometry and IL-10 ELISA (**Figure 8A**). T cells of OT-II mice express T cell receptors that are specific for chicken ovalbumin 323–339 peptide in the context of I-Ab. Expansion of T cells in this assay gives evidence that PSA-OVA SMPs were taken up by DCs, processed, and successfully presented to T cells to induce their activation. When T cells are activated, they increase in size due to metabolic processes and can be visualized by an increase in forward scattering (FSC-A) on flow cytometry. Correspondingly, only treatments that included OVA (PSA-OVA SMPs, PSA+OVA, OVA, and LPS+OVA) had FSC-A^hi^ populations (**Figure 8B**). OVA peptide presentation is necessary for TCR activation and subsequent proliferation of OT-II CD4+ T cells, so the presence of the FCS-A^hi^ population in PSA-OVA SMPs treated cells but not the blank PSA SMPs strongly suggests that OVA within the PSA-OVA SMPs was delivered, processed and presented to T cells. Moreover, >90% of FSC-A^hi^ cells are also positive for the activation marker CD25, supporting our interpretation that they are activated T cells (**Figure 8C**). In addition to T cell activation, PSA and PSA SMPs also induce the tolerogenic markers CD39 and ICOS when OVA is also present. For instance, PSA-OVA SMPs and soluble PSA+OVA increased the expression of these two markers, but PSA and unloaded PSA SMPs failed to show a similar upregulation. CD39 appears to be specifically upregulated by PSA when antigen is present (**Figure 8D**), however, ICOS upregulation is seemingly dependent on the presence of OVA rather than PSA (**Figure 8E**). T cells expressing both CD39 and ICOS were significantly increased by PSA-OVA SMPs and PSA+OVA, suggesting that this co-expression may be dependent on the simultaneous presence of both components (**Figure 8F**). Interestingly, PSA-OVA SMPs and PSA+OVA treatments also induced the greatest secretion of IL-10 (**Figure 8G**). Taken altogether, these data suggests that OVA-loaded PSA SMPs have nearly identical capacity to activate and induce tolerogenic phenotypes in OT-II T cells than soluble PSA and OVA together.

To assess whether PSA-OVA SMPs maintain their immunomodulatory abilities after being exposed to SGF and nitric oxide, we incubated PSA-OVA SMPs in gastric fluid (PSA-OVA SMPs_GF) or nitric oxide (PSA-OVA SMPs_NO) for 80 minutes prior to performing the lymphocyte reaction. All OVA containing treatment groups lead to the activation of T cells, indicating that OVA-derived peptides are still presentable to T cells even after SGF or nitric oxide treatment (**Figure 8H**). As expected, PSA-OVA SMPs were able to resist SGF exposure and induce IL-10 production to the same extent of untreated PSA-OVA SMPs (**Figure 8I**). Interestingly, although PSA-OVA SMPs_NO activated OT-II cells, it failed to induce IL-10 production and thus behaves similarly to soluble OVA (**Figure 8I**). This suggests that the PSA surrounding OVA in the PSA-OVA SMPs was completely degraded by the nitric oxide treatment, leaving behind aggregates of OVA that could still activate OT-II cells but without tolerogenic-inducing properties supplied by intact PSA. Taken together, these data shows that gastric-stable PSA-OVA SMPs can deliver presentable antigen to DCs and induce downstream tolerogenic responses in cognate T cells, thereby illustrating their potential to augment OIT.

## 4 Discussion

Current applications of OIT are limited by safety and efficacy concerns. Herein, we demonstrate that DCs treated with OVA-loaded PSA SMPs mediate the expansion of OVA-specific Treg cells *in vitro* while possessing the physiochemical characteristics necessary for oral delivery and mucus penetration. Having both PSA and protein packaged in a single sub-micron particle may be extremely efficacious *in vivo* as it will ensure that immune cells interact with both components simultaneously, potentially optimizing the induction of antigen-specific Treg cells.

Our novel water-in-oil emulsion approach with glutaraldehyde crosslinking produced sub-micron particles with physiochemical properties that are ideal for oral immunotherapy. When forced into nanodroplets within the emulsion, we hypothesize that glutaraldehyde forms covalent bonds between PSA and OVA, and between the separate molecules themselves, forming a stable sub-micron particle. Notably, past groups have fabricated effective hypoallergens using glutaraldehyde crosslinking as a method to alter protein folding to mask IgE binding sites. Thus, our fabrication method has the inherent benefit of forming hypoallergens within the PSA sub-micron particle that could retain the peptides necessary for presentation but omits binding sites for allergen-specific IgE antibodies, thereby strengthening the safety of the therapy. In addition, studies indicate that neutral or negative particles with a small diameter (<500nm) can penetrate the porous and negatively charged mucus layer of mammalian intestines (19). The PSA particles fabricated have a hydrodynamic diameter that is less than 500 nm and carry a negative surface charge which are desirable qualities for mucus penetration. However, it is likely that even smaller SMPs would be more efficient at penetrating the mucus layer of the gut. In addition, we observed that PSA-OVA SMPs have less negative zeta potential than PSA SMPs, which may indicate that the OVA may be changing or masking the particle’s surface charge. A more negative zeta potential may be achieved through different cross-linking agents or by adjusting the stoichiometric amounts of the reagents used to fabricate the particles. These parameters will be further investigated as the formulation is optimized (19). Nevertheless, the PSA-SMPs are highly stable in harsh gastric conditions and will likely reach the intestines largely intact. Collectively, the data suggests that the chemical properties of PSA sub-micron particles are conducive for oral administration and antigen processing in the phagolysosome of DCs.

The immunomodulatory capacity of OVA-encapsulated PSA sub-micron particles was tested *in vitro* using a TLR2 reporter system, fBMDCs, and co-culture experiments with fBMDCs and OVA-specific CD4 T cells. We used FLT3-L instead of GM-CSF because prior studies indicated that GM-CSF/IL-4 BMDCs are equivalent to DCs that emerge after inflammation, whereas fBMDCs better represent steady-state resident DCs and induce higher number of plasmacytoid DCs (pDCs) (20). Recent studies indicate pDCs are more potent responders to PSA in the gut than conventional DCs and induce higher IL-10 production in T cells (20). OVA antigen was used as a proxy for allergens to exploit OT-II mice since no TCR transgenic mice exists for more common food allergens, such as those derived from peanut and shrimp.

Although the PSA+OVA, PSA-OVA SMPs, and LPS+OVA groups all increased CD86, MHCII, and ICOSL expression in DCs and robustly activate T cells in co-culture, only the PSA+OVA and PSA-OVA SMPs induced tolerogenic T cells, as characterized by CD25, CD39, ICOS, and IL-10 upregulation. This suggests that other ligand-receptor interactions, in addition to CD86, MHCII, and ICOSL, are involved in PSA-mediated tolerance. This is not unexpected, for CD86 and ICOSL has also been measured in pro-inflammatory contexts, which illustrates the complexity of PSA immunomodulation and warrants further investigation into its mechanisms. In addition, although IL-10 is produced by LPS+OVA in our co-culture experiments, it is done so in significantly lower amounts when compared to PSA-OVA SMPs and PSA+OVA treatment groups, which indicates that mechanism in which LPS induces IL-10 is fundamentally different from PSA. Prolonged LPS exposures likely activates a negative feedback loop that caps immune activation through basal IL-10 production. Contrastingly, PSA actively tolerizes T cell responses to induce copious amounts of IL-10. Importantly, PSA-OVA SMPs can induce tolerogenic T cells to the same extent as soluble PSA+OVA, which shows that antigen presentation and immunomodulation is not hindered by SMP fabrication. Of the T cell markers measured, CD39 appears to be the strongest predictor of IL-10 production, and CD39 will likely become a more useful marker for PSA-mediated tolerance when compared to FoxP3 due to the previous observation that PSA upregulates FoxP3^+^CD39^+^ and FoxP3^-^CD39^+^ populations that can both produce IL-10 (21). Thus, PSA-trained T cells can produce IL-10 irrespective FoxP3 but are seemingly dependent on CD39 expression. Finally, we showed that the modulatory properties of PSA-SMPs are still maintained after 80-minute incubation in SGF.

Our series of *in vitro* experiments could have been bolstered by the inclusion of a more robust panel DC and T cell markers and chemokines recently discovered through RNASeq, including Dectin-1, CXCL10, Tim3, PD1 and FoxP3, which have been shown to be upregulated after PSA exposure (10, 22). Moreover, it would be informative to perform RNASeq analysis on T cells after co-culture with PSA-OVA SMPs to assess PSA-directed T cell modulation in the context of antigen delivery and presentation. Furthermore, the efficacy of the PSA SMPs will be more robustly scrutinized if challenged in an *in vivo* model of food allergy, for an animal model would better recapitulate the complexities of the immune system compared to *in vitro* assays. Although we have made tremendous progress illustrating the potential of PSA SMPs for tolerogenic delivery of antigen, more work is needed to fully characterize their immunomodulatory capabilities and to realize their potential to augment oral immunotherapy.

## 5 Conclusions

In recent years, investigations linking the gut microbiome to the pathogenesis of food allergy have led investigators to explore whether immune tolerance could be established by restoring microbial dysbiosis through fecal microbiota transplant or oral probiotics. Although promising, methods involving the introduction of live microbes are often limited to non-fastidious microbes that may not be the most impactful immunomodulators of the gut microbiome. Thus, the adaptation of commensal-derived molecules, such as PSA, into novel therapies may circumvent this major bottleneck. To our knowledge, we are the first group to successfully utilize PSA, a commensal-derived polysaccharide, as a biomaterial for the fabrication of tolerogenic particles. We hope the work illustrated here compels further investigation into biomaterial-based strategies that combine imaginative delivery platforms with immunomodulatory cues from commensal organisms. This holistic approach may be the panacea to numerous inflammatory disorders, including food allergies.

## Data Availability

The datasets presented in this article are not readily available due to consideration for patentability. Requests to access the datasets should be directed to jlewis@bme.ufl.edu.
