# Supplementary material for: Gut commensal bacteria-derived polysaccharide sub-micron particles induce antigen-specific, tolerogenic responses
Source: Front Immunol. 2025 Sep 25;16:1599480. doi: 10.3389/fimmu.2025.1599480 (PMC12507582; doi:10.3389/fimmu.2025.1599480)
Supplement: Supplementary file 1 [file Supplementaryfile1.docx]

**Supplementary Data**


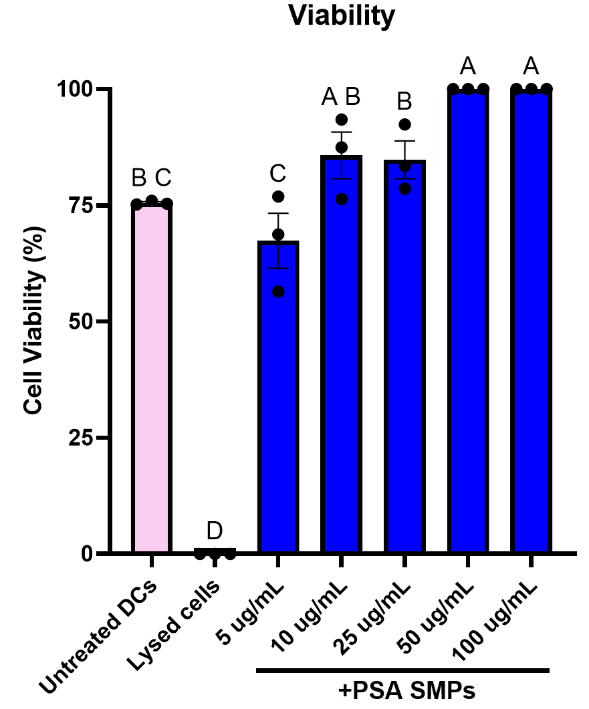


**Supplementary Figure 1.** Viability of fBMDCs treated with PSA SMPs. fBMDCs were seeded at 1.25 x 10^4^ cells per well into 96-well plate and treated with PSA SMPs at 5, 10, 25, 50, and 100 ug/mL doses for 48 hours. After 48 hours, LDH assay was performed. Treatment groups sharing a letter are not significantly different, whereas groups with different letters are (p<0.05). All statistical analyses were one-way ANOVAs corrected for multiple comparisons Tukey’s test via GraphPad Prism 9.
